# Unraveling the relationship among lifestyle, tumor eruption site, histopathological grading, and oral squamous cell carcinoma: A clinicopathological study

**DOI:** 10.12669/pjms.42.3.13124

**Published:** 2026-03

**Authors:** Quratulain Javaid, Tasneem Fatima, Jehan Alam, Muhammad Faraz Anwar

**Affiliations:** 1 Quratulain Javaid, MPhil-Anatomy. Professor, Anatomy Department, Bahria University Health Sciences, Karachi, Pakistan; 2 Tasneem Fatima, PhD. Professor, Anatomy Department, Bahria University Health Sciences, Karachi, Pakistan; 3 Jehan Alam, FCPS. Professor, Department of Oral Surgery, Jinnah Postgraduate Medical Centre, Karachi, Pakistan; 4 Muhammad Faraz Anwar, PhD. Assistant Professor Biochemistry Department, Bahria University Health Sciences, Karachi, Pakistan

**Keywords:** Histopathological Grading, Lifestyle, Oral Squamous Cell Carcinoma, Tumor Site

## Abstract

**Objective:**

This study aimed to determine the relationship among lifestyle practices, histopathological grading, and tumour site in oral squamous cell carcinoma (OSCC) patients and to assess their effect on clinicopathological outcomes.

**Methodology:**

The cross-sectional descriptive study was conducted at JPMC and PNS Shifa Hospitals, Karachi comprising 107 OSCC patients diagnosed between September 2024 to August 2025. Data on risk factors, tumor eruption sites and grading of tumor were documented. The clinicopathological outcomes included TNM staging and mortality of patients during period of study. Associations between clinicopathological variables and outcomes were assessed using chi-square / Fisher’s exact test. Survival analysis was performed by Kaplan-Meier method.

**Results:**

Most common tumor site was buccal mucosa 62(57.9%). Areca nut was the frequent most consumed smokeless tobacco 69 (64.4%). A significant association was found between lifestyle risk factors and staging. Trend analysis documented a progressive shift toward advanced stage with increasing grade. The estimated mean survival time was longest for Grade-I tumors (336 days).

**Conclusion:**

Smokeless tobacco in form of areca nut was the most prevalent risk factor and buccal mucosa the most common location of cancer. The lifestyle risk factors showed significant association with staging. Increasing grade was found to decrease the survival.

## INTRODUCTION

Globally, among malignancies of head and neck region, the oral squamous cell carcinoma (OSCC) is most prevalent. It has comparatively higher incidence in South Asia especially Pakistan and contributes to substantial burden on health sector.^1^ OSCC is associated with poor survival outcomes because of diagnosis at late stages. This contributes to decrease in survival rate and increased mortality. When detected early, five-years survival rate can be 80% as compared to 30% when diagnosed late.^2^

Various factors including the patterns of life style, tooth cleaning, site of tumor and deviation from normal histopathological morphology contributes to clinicopathological outcomes.^3^ Tobacco both smoked and smokeless along with alcohol consumption is considered to be playing a significant role. Constant exposure of carcinogens leads to various disruptions at gross as well as cellular levels including irritation of mucosa, genetic makeup abnormalities and dysplastic changes. In Pakistan, smokeless tobacco play a vital role in OSCC development.^1^,^4^ In Pakistan where alcohol is religiously looked down upon, still it is among the cancer-causing factors. Combination of all of these elements contributes towards high incidence of OSCC.^5^,^6^ OSCC prognosis is also dependent on histopathological grading and well differentiated cancers have favourable outcomes as compared to poorly differentiated ones.^7^

An important contributor to cancer progression is its location. Shinohara et al documented that buccal tumors have less recurrence rate as compared to other regions of oral cavity. When five-years survival rate was compared, other region tumors had slightly better survival (82.5%) than buccal region cancers (80.6%). Stage IV cancers of buccal region had poor outcomes (38.1%) as compared to other regions’ stage IV cancers (62.9%).^8^ In Taiwan, high mortality was observed in hard palate and alveolar ridge tumors.^9^

The current study aimed to determine relationship among lifestyle practices, histopathological grading, and tumour site in OSCC patients and to assess their effect on clinicopathological outcomes. The rationale for the current study stems from the clinical gap in the regional literature; the clinical requirement to comprehend in what way lifestyle practices directly influence anatomical tumor sites and subsequent histological aggressiveness. The study is anticipated to shed light on the role of these factors contributing towards development of OSCC. Though previous studies have documented the linkage between lifestyle factors and OSCC or tumor site or histopathological grading in isolation but their combined influence remains under-reconnoitred. Explicitly, there is a deficiency of longitudinal data with respect to the Pakistani perspective utilizing Kaplan-Meier analysis to correlate histopathological grading of OSCC with 12 months overall survival (OS). The evaluation of these multifactorial variables provides a unique prognostic context that evaluates how tumor aggressiveness influences survival of patients in the Karachi population.

## METHODOLOGY

This prospective-longitudinal study was conducted at Jinnah Postgraduate Medical Centre and PNS Shifa Hospital Karachi from September 2024 to August 2025. Non-randomised purposive sampling was used. Sample size was calculated by Openepi version 3 calculator.^10^ We used expected frequency of 35% for the primary outcome (histopathological grade). With 95% confidence level (Z=1.96), and a precision (d) of 9.5%, the minimum sample size was 96 but we included 107 to balance possible drop-outs or loss to follow up.

### Inclusion and exclusion Criteria:

Patients aged 19-78 years diagnosed on histopathological biopsy results with no prior history of cancer other than OSCC were included. Those who had received chemotherapy and radiotherapy were excluded. All participants provided informed consent. A standardized questionnaire was used to document the demographic factors, tumor location and lifestyle factors. To determine survival status of patients, a follow-up survey was performed in September 2025. Survival period was defined as time interval from date of recruitment to date of last follow-up or death, whichever occurred first. Patients who were alive at the last follow-up were considered satisfactory.

### Operational Definitions:

a) ***Histological grading of OSCC:*** The histological grading was classified according to degree of differentiation: well-differentiated, moderately differentiated, and poorly differentiated. The three tier grading is according to WHO classification.^3^-^7^
b) ***Overall survival:*** Based on regional studies, overall survival was described as the time duration from preliminary diagnosis to demise or the completion of the 12 months study duration.^3^,^8^,^9^ The survival status of patients was recorded at the completion of 12 months period.

### Ethical approval:

Study was approved by Institutional Review Board of Bahria University Health Sciences (BUHS-IRB# 068-/24; dated: May 27, 2024).

### Statistical analysis:

Descriptive statistics were used to summarize demographic characteristics and distribution of risk factors. Continuous variables were presented as means with standard deviations, while categorical variables were presented as percentages. To determine association between categorical variable, Chi-square test/Fisher’s exact test was used. To determine trends across ordered categories, Linear-by-Linear Association (trend analysis) was used. Survival analysis was performed by Kaplan-Meier method. A p-value of <0.05 was considered statistically significant. Statistical analysis was performed using SPSS version 27.

## RESULTS

Mean age of patients was 50.5±10.7 years. There were 82 males and 25 females. Of the 107 patients, 90 (84.1%) had history of smokeless tobacco use, 40 (37.4%) and 24 (22.4%) were involved in consumption of smoking and alcohol respectively. Of those who consumed smokeless tobacco, majority consumed areca nut 69 (64.4%) followed by betel quid 48 (44.8%), naswar 42 (39.2%), tobacco 42 (39.2%), mawa 18 (16.8%) and other 18 (16.8%) while combination of smokeless tobacco was used by 61 (57%). Clinicopathological characteristics of patients including tumor site, histopathological grade, and clinical stage, are summarized in Table-I.

**Table-I:** Clinicopathological characteristics of Oral Squamous Cell Carcinoma (OSCC) patients.

Tumor eruption sites		
Site	Frequency (N)	Percentage %
Buccal mucosa	62	57.9
Alveolar region	17	15.9
Tongue	11	10.3
Lip	11	10.3
Palate	6	5.6
*Histopathological Grading*		
*Grade*	*Frequency (N)*	*Percentage %*
I	31	28.9
II	58	54.2
III	18	16.8
*Clinical Stage at Diagnosis*		
*Stage*	*Frequency (N)*	*Percentage %*
I	16	15.0
II	14	13.1
III	21	19.6
IV	56	52.3
*TNM staging*		
*(Tumor size)*		
*T category*	*Frequency (N)*	*Percentage %*
T1	26	24.3
T2	34	31.8
T3	13	12.1
T4	34	31.8
*(Nodal involvement)*		
*N category*	*Frequency (N)*	*Percentage %*
N0	31	29
N1	18	16.8
N2a	6	5.6
N2b	32	29.9
N2c	6	6.6
N3	14	13.1
*(Metastasis)*		
*M category*	*Frequency (N)*	*Percentage*
M0	101	94.4
M1	6	5.6

T: tumor size (T1 = ≤2 cm, T2 = >2-4 cm, T3 = >4 cm, T4 = invasion into adjacent structures). N: nodal involvement (N0: No regional lymph node metastasis; N1-N3 shows metastasis. N1: single-nearby lymph node ≤3cm. N2a: single-nearby lymph node (same side), 3-6cm. N2b: multiple-nearby lymph node(same side), ≤6cm. N2c: ≤6cm-both sides of neck, N3: Metastasis in a lymph node >6cm) M: Distant metastasis (M0 = absent, M1 = present).

There was a significant association between lifestyle-related risk factors and clinicopathological outcome like tumor stage (Table-II). A significant association was found between histological grade and clinical stage of tumor. Trend analysis confirmed a progressive shift toward advanced stage of disease with higher grade (Table-III).

**Table-II:** Effect of risk factors on staging of tumor.

Staging of OSCC						
Type of risk factors	I	II	III	IV	Total	P value
***Smokeless tobacco***						0.002*
Yes	16 (17.8%)	8 (8.9%)	21 (23.3%)	45 (50%)	90 (100%)	
No	0 (0%)	6 (35.3%)	0 (0%)	11 (64.7%)	17 (100%)	
Total	16 (15%)	14 (13.1%)	21 (19.6%)	56 (52.3%)	107 (100%)	
***Smoking***						0.058*
Yes	3 (7.5%)	3 (7.5%)	12 (30%)	22 (55%)	40 (100%)	
No	13 (19.4%)	11 (16.4%)	9 (13.4%)	34 (50.7%)	67 (100%)	
Total	16 (15%)	14 (13.1%)	21 (19.6%)	56 (52.3%)	107 (100%)	
***Alcohol***						0.005*
Yes	0 (0%)	3 (12.5%)	10 (41.7%)	11 (45.8%)	24 (100%)	
No	16 (19.3%)	11 (13.3%)	11 (13.3%)	45 (54.2%)	83 (100%)	
Total	16 (15%)	14 (13.1%)	21 (19.6%)	56 (52.3%)	107 (100%)	
***Tooth Brushing***						0.011*
Yes	6 (9.5%)	12 (19%)	16 (25.4%)	29 (46%)	63 (100%)	
No	10 (22.7%)	2 (4.5%)	5 (11.4%)	27 (61.4%)	44 (100%)	
Total	16 (15%)	14 (13.1%)	21 (19.6%)	56 (52.3%)	107 (100%)	

Chi-square test was applied. For categories with small cell counts (expected <5), Fisher’s exact test was used. Yes = used; No = not used

* p<0.05, **p<0.001.

**Table-III:** Association of grading and staging of OSCC patients.

	Stage					
Grade	I	II	III	IV	Total	P value
I	8 (25.8%)	2 (6.5%)	12 (38.7%)	9 (29%)	31 (100%)	0.000
II	8 (13.8%)	12 (20.7%)	6 (10.3%)	32 (55.2%)	58 (100%)	
III	0 (0%)	0 (0%)	3 (16.7%)	15 (18.3%)	18 (100%)	
Total	16 (15%)	14 (13.1%)	21 (19.6%)	56 (52.3%)	107 (100%)	

Chi-square test was applied. For categories with small cell counts (expected <5), Fisher’s exact test was used. *p<0.05, **p<0.001.

**Fig.1:**
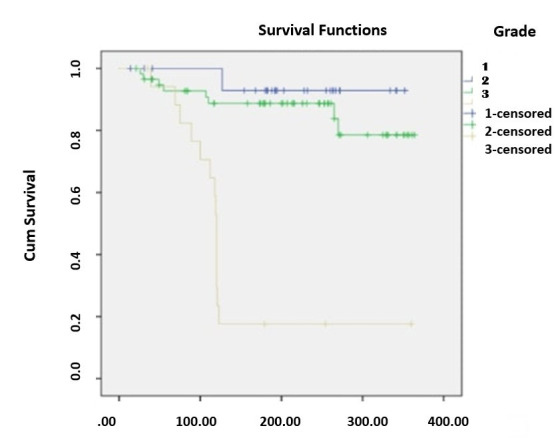
Kaplan-Meier survival curves for overall survival stratified by histological grade of OSCC during a 12 months follow-up period.

Kaplan-Meier survival analysis demonstrated a statistically significant difference in overall survival among histological grades (p < 0.001). The estimated mean survival time was longest for Grade-I tumors (336 days), followed by Grade-II (320 days), and shortest for Grade-III (297 days). For Grade-I, two events and 29 censored cases were recorded out of 31; for Grade-II, eight events and 50 censored out of 58; and for Grade-III, 14 events and four censored out of 18 (Fig.1).

## DISCUSSION

Our data reported more smokeless tobacco cases than smoked while a few consumed alcohol. Parallel findings were reported in South Asia where smokeless tobacco consumption was on rise.^11^,^12^ Type of smokeless tobacco varies geographically. Our study reported areca nut to be the determinant. Similarly a Karachi based study has also documented areca nut to be root cause^13^ whereas a study conducted in India has mentioned gutka and manpuri to be most common contributing factors.^12^ Mehrotra et al have mentioned gutka along with oral snuff while Yasin et al have also mentioned gutka followed by betel quid and naswar to be the abortifacient factors.^11^,^14^ Contrary to high prevalence of smokeless tobacco in our study, a research conducted in Northern Pakistan has mentioned alcohol to be commonest cause followed by paan, betel quid and naswar while smoked tobacco was among least commonest causative agent.^6^ Yasin et al mentioned that only 6% were involved in smoking while only 1% alcoholics contributed to OSCC.^14^ The discrepancy in risk factors differences reflects regional variances.

With respect to site of cancer, our study found buccal cancer to be most commonly involved while a study in Lahore mentioned tongue followed by buccal mucosa.^15^ Dviwedi et al in In India reported tongue followed by buccal mucosa among prevalent sites of OSCC.^12^ A study conducted in Finland documented tongue followed by gingivae to be more commonly involved.^16^ It could be postulated that consumption of different forms of risk factors are linked to different sites of cancers in oral cavity due to exposure of carcinogenic component of involved recreational substances.

The current study observed a significant relation between risk factors and staging of OSCC. This is in line with the studies conducted in other regions of the world, highlighting the importance of carcinogenic components disrupting the normal anatomy.^17^-^20^ Saleem et al documented gutka to be most common reason for invasive OSCC.^21^ Another study conducted in Hyderabad, India stated the odd’s ratio of 2.8 with heavy tobacco consumption and well-differentiated histopathological grading while the odd’s ratio was reported to be nine with poorly differentiated oral tissues.^22^ Eloranta et al. documented the association of tobacco and alcohol with oral cancer formation.^16^

A study reported that 15.7% OSCC patients had no association with any smokeless or smoked form of neither tobacco nor alcohol.^14^ Genetic predisposition can be reasons for cancer development in patients who have no history of exposure to risk factors.^1^ Tooth cleaning practices have a great impact on the growth and staging of OSCC. Our study showed a significant correlation between improper cleaning routine and high stage. Analogous results were mentioned by a study conducted in North-West of Pakistan implying that poor hygiene was a strong predictor of OSCC.^17^ A study conducted in Jinan, China has mentioned that lack of oral hygiene is associated with advanced staging of OSCC.^18^ The reason could be that inadequate cleaning causes inadequate removal of plaque, therefore exacerbates vulnerability of oral mucosal environment.

The present study found an association between grading of OSCC and staging. Analogous results were mentioned by a study conducted in Rawalpindi, Pakistan.^3^ This shows that poorly differentiated tumors are associated with advanced disease clinical presentation. The current study showed association between high grade of OSCC and overall survival. Parallel results were mentioned by studies conducted in Karachi, Pakistan, Spain and Taiwan.^23^-^25^ The reason could be that high grade is associated with invasive tumor that can metastasise leading to short survival.

### Strengths:

A major strength of this study is that we used a prospective-longitudinal follow-up design. It incorporated Kaplan-Meier survival curves to determine correlation between histopathological grading and overall survival for 12-month duration. Short-term survival analysis presented preliminary discernments into progression of disease and associated mortality. Employing this design helps us have a dynamic understanding of disease progression which was not possible had we used cross-sectional design. Also, study was conducted at tertiary care hospitals in Karachi that cater patients from general population especially the low socio-economic status individuals that are associated with burden of disease in our setup. This strategy makes our results representative of Karachi burden of OSCC.

### Limitations:

As sample size was small, therefore findings of study cannot be generalised. As the survival analysis was based on a period of 12 months, therefore long-term survival analysis could not be evaluated.

## CONCLUSION

Smokeless tobacco in form of the areca nut was the most prevalent risk factor and buccal mucosa the most common location of cancer. The lifestyle risk factors showed significant association with staging. Increasing grade caused decrease in the survival span.

### Recommendations:

Extended Longitudinal studies should be conducted so long-term outcomes can be determined. Additionally, targeted molecular analysis of the NOTCH1 gene should be performed to identify possible genetic predispositions.

### Authors` Contribution:

**QJ:** Concept of work, acquisition & analysis of data, drafting manuscript, critical revision, final approval and Agreement to be accountable for all aspects of the work

**TF:** Concept of work, critical revision, final approval

**JA:** Concept of work, acquisition of data

**MFA:** Concept of work, critical revision, final approval.
